# CircRNA circRNA_102171 promotes papillary thyroid cancer progression through modulating CTNNBIP1-dependent activation of β-catenin pathway

**DOI:** 10.1186/s13046-018-0936-7

**Published:** 2018-11-13

**Authors:** Wen Bi, Jiayu Huang, Chunlei Nie, Bo Liu, Guoqing He, Jihua Han, Rui Pang, Zhaoming Ding, Jin Xu, Jiewu Zhang

**Affiliations:** 1 Department of Head and Neck Surgery, The Third Affiliated Hospital of Harbin Medical University, No.150 Haping Road, Nangang District, Harbin, 150081 China; 2 Department of Pediatrics, The First Affiliated Hospital of Harbin Medical University, Harbin, 150070 China; 3 Department of Cell Biology, Harbin Medical University, No.157 Baojian Road, Nangang District, Harbin, 150081 China

**Keywords:** Papillary thyroid cancer, Circular RNA, circRNA_102171, CTNNBIP1, Wnt/β-catenin

## Abstract

**Background:**

As a type of recently discovered noncoding RNA, circular RNAs (circRNAs) exert pivot biological functions in diverse cancers. However, the role of circRNA_102171 in papillary thyroid cancer (PTC) has not been investigated. Our study was focused on the functional investigation toward circRNA_102171 in PTC progression. And we also aimed to reveal its potential molecular mechanism.

**Methods:**

The expression pattern of circRNA_102171 was determined using quantitative polymerase chain reaction (qPCR) in PTC samples and cell lines. Cell proliferation was examined utilizing CCK8, colony formation and EdU incorporation assays. Apoptosis was analyzed by Annexin V/PI staining and FACS detection. Cell migration and invasion was measured using Transwell assay. Tumor growth in vivo was determined through a xenograft assay. RNA-pulldown, RNA-IP (RIP) and RNA-EMSA were used to analyze the interaction between circRNA_102171 and CTNNBIP1.

**Results:**

CircRNA_102171 expression was upregulated in tumor tissues and cell lines. CircRNA_102171 silencing suppressed PTC cell proliferation, migration and invasion while promoting apoptosis. CircRNA_102171 knockdown inhibited PTC growth in vivo. CircRNA_102171 interacted with CTNNBIP1 to block its interaction with the β-catenin/TCF3/TCF4/LEF1 complex, leading to activation of Wnt/β-catenin pathway.

**Conclusions:**

CircRNA_102171 overexpression promotes PTC progression through activating Wnt/β-catenin pathway in a CTNNBIP1-dependent way.

## Background

Thyroid cancer (TC) is the most prevalent cancer in the endocrine system whose incidence and morbidity are steadily growing around the world [1]. About 83% patients with TC belong to papillary thyroid carcinoma (PTC) [2]. The risk factors of PTC include genetic mutation and environmental exposure [3]. Most PTC patients at the early stage show a favorable prognosis after treatment with thyroidectomy and radioactive iodine [4]. However, the recurrence is greatly increased if metastasis exists [5]. Hence, it is still important to understand the molecular mechanism of PTC progression. Furthermore, developing novel therapeutic approach is urgently required.

Circular RNA (circRNA), together with microRNA (miRNA) and long noncoding RNA (lncRNA) are most explored noncoding RNAs (ncRNAs) in the past years [6]. CircRNA is characterized by a covalently closed loop and has no capacity to code protein [7]. Recent researches have proven that circRNA participates in various physiological and pathological processes [8, 9]. Especially, the roles of circRNA in tumorigenesis are widely investigated recently. For example, Chen et al. predicted that hsa_circ_0032462, hsa_circ_0028173 or hsa_circ_0005909 promotes osteosarcoma progression through regulating CADM1 expression [10]. Zeng et al. showed that circ-VANGL1 promotes bladder cancer cell proliferation, migration and invasion via miR-605-3p/VANGL1 axis [11]. Song et al. reported that hsa_circ_0007534 silencing inhibits breast cancer growth and metastasis through modulating miR-593/MUC19 pathway [12]. In PTC, the study about circRNAs has just begun. Only a few circRNAs have been investigated, such as circZFR [13] and circ-ITCH [14]. Thus, it is essential to reveal the association between circRNA expression and PTC development.

CircRNA_102171, with a length of 309 nucleotides, is derived from back-splicing of SMURF2 mRNA. The function of circRNA_102171 is unknown. In this study, we aimed to reveal its function and molecular mechanism in PTC progression. We showed that circRNA_102171 expression was elevated in PTC tissues. Functionally, circRNA_102171 promotes PTC cell proliferation, migration and invasion while suppressing apoptosis. Mechanistically, circRNA_102171 interacts with CTNNBIP1 and blocks its association with the β-catenin/TCF complex, leading to activation of Wnt/β-catenin pathway. Our study provides a novel signaling pathway involved in PTC progression.

## Methods

### Patients and tissue specimens

Forty-seven pairs of PTC tissues and normal tissues were obtained from The Third Affiliated Hospital of Harbin Medical University. This study was approved by the ethics committee of The Third Affiliated Hospital of Harbin Medical University. All written informed consents were acquired from patients. All tissues received no radiotherapy or chemotherapy before surgery and immediately stored in liquid nitrogen after surgery.

### Cell culture and transfection

PTC cell lines (TPC-1, NPA87 and KAT-5) and normal cell line Nthy-ori3–1 were from the Chinese Academy of Sciences (Shanghai, China). Cell lines were cultured in DMEM (Gibco, Carlsbad, CA, USA) containing 10% FBS (Gibco) and maintained in a humidified incubator at an atmosphere with 5% CO_2_.

Small interfering RNA (siRNA) against circRNA_102171 (si-circ; 5′-AGAGGACAGATAGTAGGACTT-3′) was designed using the circinteractome tool (https://circinteractome.nia.nih.gov/bin/) and bought from RiboBio Co., Ltd. (Guangzhou, China). siRNAs (50 nM) were transfected into TPC-1 and KAT-5 cells using Lipofectamine 3000 (Invitrogen) following the manufacturer’s instructions.

### Quantitative real-time PCR (qRT-PCR)

Total RNAs were isolated from PTC tissues and cell lines using TRIzol reagent following the manufacturer’s instructions. qRT-PCR were carried out according to a previous study [15]. All primers acquired from Sangon Biotech (Shanghai, China). GAPDH or U6 works as internal control.

### Cell viability assay

Cell viability was measured using the cell counting kit-8 (CCK-8, Sigma-Aldrich) following the manufacturer’s instructions. In brief, 2000 cells per well were seeded in 96-well plates and cultured at described days. Then CCK8 solution were added and absorbance at 450 nm were determined through a SpectraMax microtiter plate reader (Molecular Devices, Carlsbad, CA, USA).

### Colony formation assay

One thousand PTC cells per well were seeded into 6-well plates and cultured for 14 days. Then cells were fixed with methanol and stained with 0.5% crystal violet. Colonies were then counted using a Nikon Eclipse E600 microscope (Nikon Instruments, Melville, NY).

### Cell apoptosis assay

Cell apoptosis were measured using an Annexin V-FITC Apoptosis Staining/Detection kit (Cambridge, MA) according to the manufacturer’s instructions. The samples were analyzed with a FACScan flow cytometer (BD Biosciences, San Jose, CA).

### Transwell assay

Cell migration and invasion was analyzed using the transwell chamber (8 μ pore size; Corning Incorporated, Corning, NY, USA). In brief, 2 × 10^4^ PTC cells in 200 μl serum-free DMEM were seeded into the upper chamber pre-coated with Matrigel matrix (BD Biosciences, Franklin Lakes, NJ, USA) for invasion analysis. The lower chamber was filled with 600 μm complete DMEM medium. After incubation for 48 h, the cells in the upper chamber were removed. And cells in the down chamber were fixed and stained with 0.5% crystal violet (Beyotime Institute of Biotechnology, Jiangsu, China). Cell number was counted using a light microscope.

### RNA IP

RNA IP was utilized to determine endogenous interaction between circRNA_102171 and CTNNBIP1. In brief, PTC cell lysates were incubated with anti-CTNNBIP1 for 4 h at 4 °C. Then beads were added and incubated for another 2 h. The precipitated RNAs were eluted and analyzed by qRT-PCR.

### Animal study

Xenograft assay was used to analyze the role of circRNA_102171 in vivo. Animal assay was approved by the ethics committee of The Third Affiliated Hospital of Harbin Medical University. In brief, 1 × 10^7^ shcircRNA_102171 or control TPC-1 cells were subcutaneously injected into the flanks of nude mice from Charles River (Beijing, China). At described times, tumor volumes were measured. 25 days later, tumor weights were analyzed.

### RNA electrophoretic mobility shift assays (RNA-EMSA)

RNA-EMSA was performed using the LightShift Chemiluminescent RNA EMSA Kit (Thermo Scientific) according to a previous study [16].

### Statistical analysis

Statistical analysis was completed using SPSS software (version 22.0, SPSS Inc., Armonk, NY, USA). Statistical significance was measured using Student’s t-test or the ANOVA test. Each result from at least three independent experiments was displayed as mean ± standard deviation (SD). *p* < 0.05 was considered as statistical significance.

## Results

### CircRNA_102171 is upregulated in PTC

**Fig. 1:**
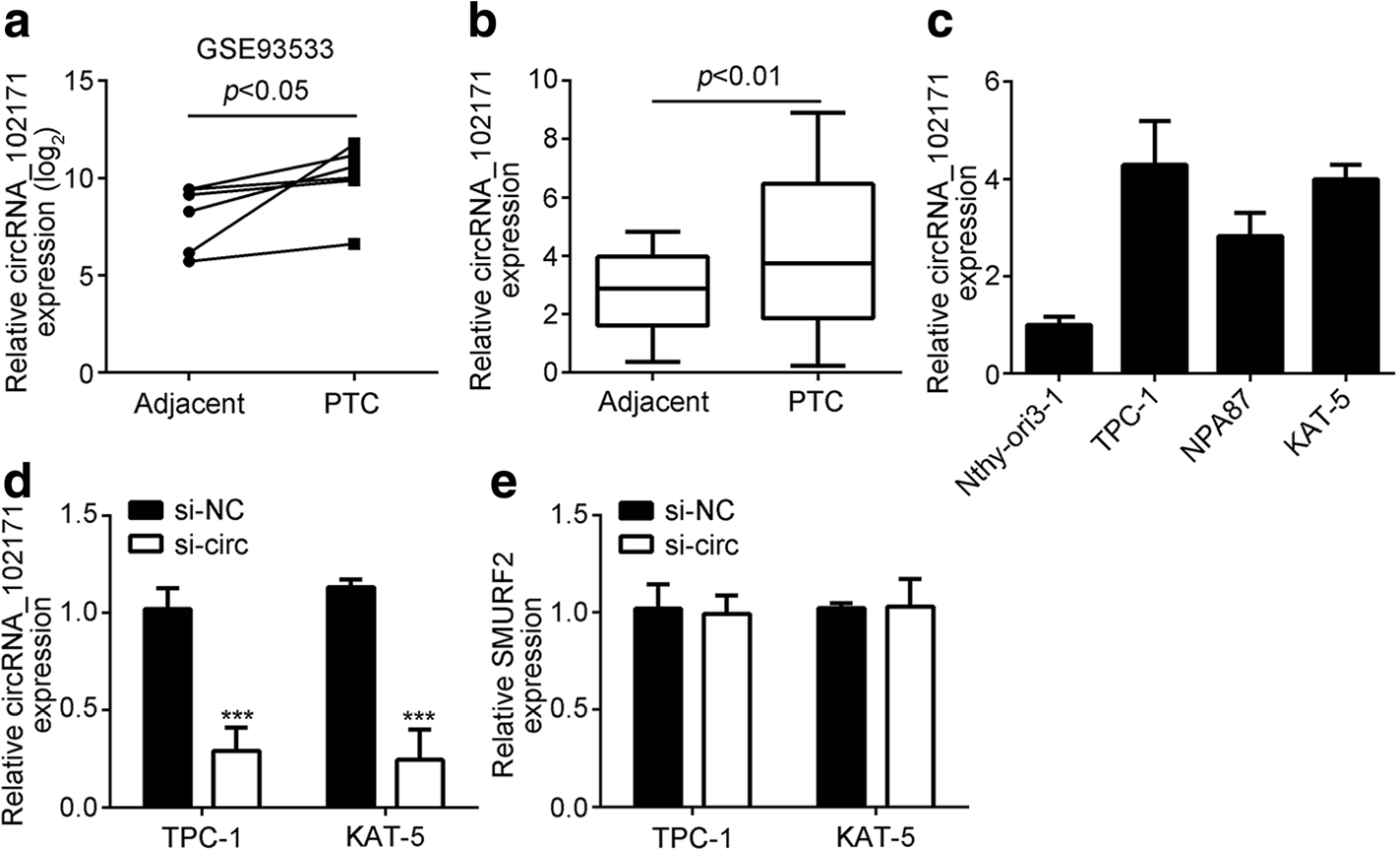
CircRNA_102171 is upregulated in PTC. **a** According to a public dataset (GSE93533), circRNA_102171 expression was upregulated in PTC tissues. **b** Through qRT-PCR, circRNA_102171 level was increased in PTC tissues (*n* = 47) compared to adjacent normal tissues (*n* = 47). **c** CircRNA_102171 level was elevated in PTC cell lines by qRT-PCR. **d** qRT-PCR showed decreased level of circRNA_102171 in TPC-1 and KAT-5 cells after transfection with siRNA. Si-NC: siRNA negative control. si-circ: siRNA targeting circRNA_102171. **e** qRT-PCR showed circRNA_102171 silencing did not affect the expression of SMURF2 in TPC-1 and KAT-5 cells. Si-NC: siRNA negative control. si-circ: siRNA targeting circRNA_102171. ***p < 0.001

Firstly, in order to investigate the relationship between circRNA and PTC, we analyzed the online dataset (GSE93522). According to this data, we found that circRNA_102171 is the most upregulated circRNA in PTC tissues compared to normal control tissues. Thus, we chose it to further investigation. As shown, the expression of circRNA_102171 was significantly upregulated in all five PTC tissues compared to matched normal tissues (Fig. 1a). Its level was also markedly upregulated in collected 47 PTC tissues compared to their corresponding adjacent normal tissues (Fig. 1b). Similarly, circRNA_102171 level was also higher in PTC cell lines, including TPC-1, NPA81 and KAT-5 cells than that in Nthy-ori3–1 cells (Fig. 1c). Because circRNA_102171 level was the highest in TPC-1 and KAT-5 cells (Fig. 1b), we knocked circRNA_102171 down in these two cell lines using specific siRNAs (Fig. 1d). Notably, circRNA_102171 silencing did not affect the expression of its linear mRNA SMURF2 (Fig. 1e).

### CircRNA_102171 silencing inhibits proliferation and promotes apoptosis

**Fig. 2:**
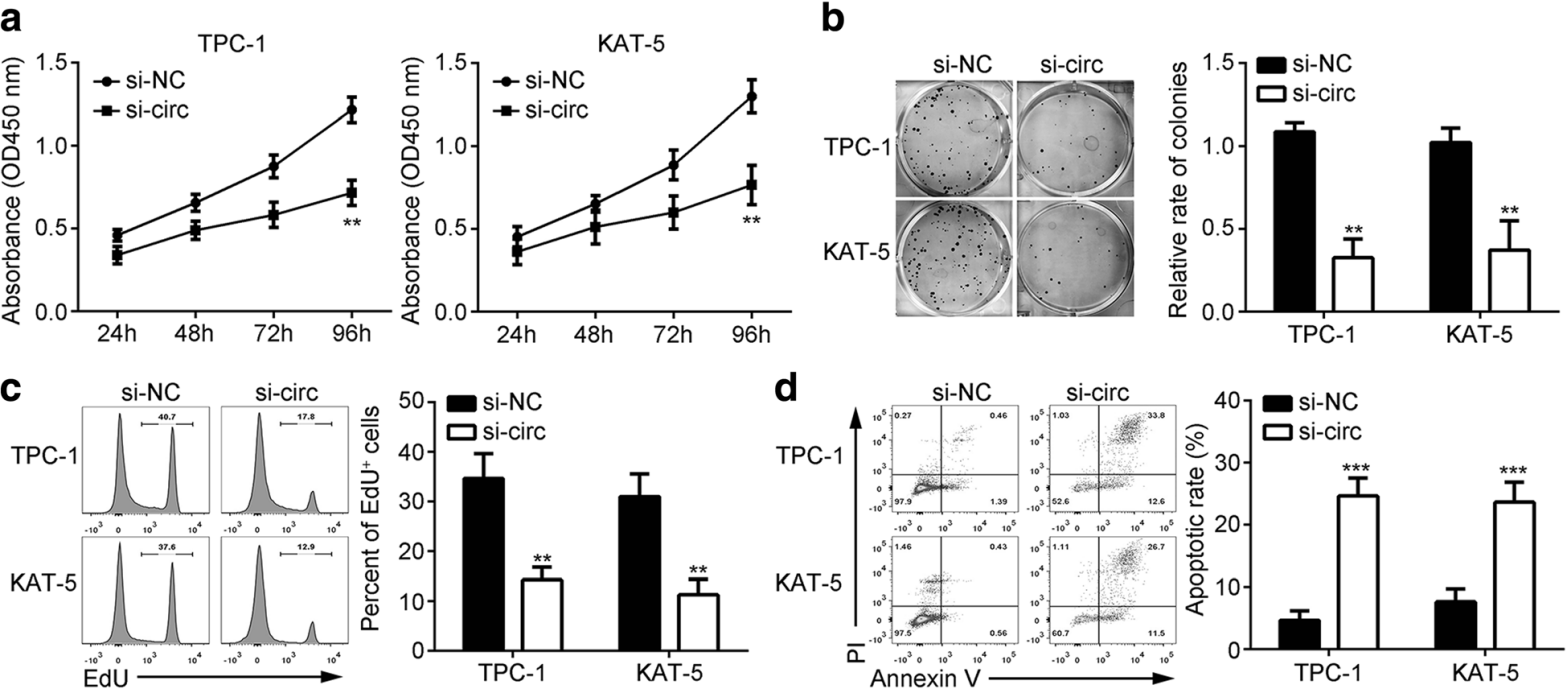
CircRNA_102171 silencing inhibits proliferation and promotes apoptosis. **a** According to CCK8 assay, circRNA_102171 depletion inhibited PTC cell proliferation. **b** CircRNA_102171 silencing decreased colony number. **c** Decreased rate of EdU positive PTC cells after circRNA_102171 knockdown. **d** CircRNA_102171 knockdown increased apoptotic PTC cells. Si-NC: siRNA negative control. si-circ: siRNA targeting circRNA_102171. **p < 0.01 and ***p < 0.001

To analyze the potential function of circRNA_102171, we performed functional experiments. CCK8 assay showed that circRNA_102171 silencing significantly suppressed proliferation of TPC-1 and KAT-5 cells (Fig. 2a). Colony formation assay also indicated that circRNA_102171 knockdown decreased colony number (Fig. 2b). Moreover, in vitro EdU incorporation assay showed that circRNA_102171 depletion significantly inhibited the division of TPC-1 and KAT-5 cells (Fig. 2c). Above results support that circRNA_102171 is important for PTC cell proliferation. Additionally, the effect of circRNA_102171 on apoptosis was examined by FACS. Results showed that circRNA_102171 knockdown increased the apoptotic cell rate dramatically (Fig. 2d).

### CircRNA_102171 knockdown suppresses migration and invasion, and reduces tumor growth in vivo

**Fig. 3:**
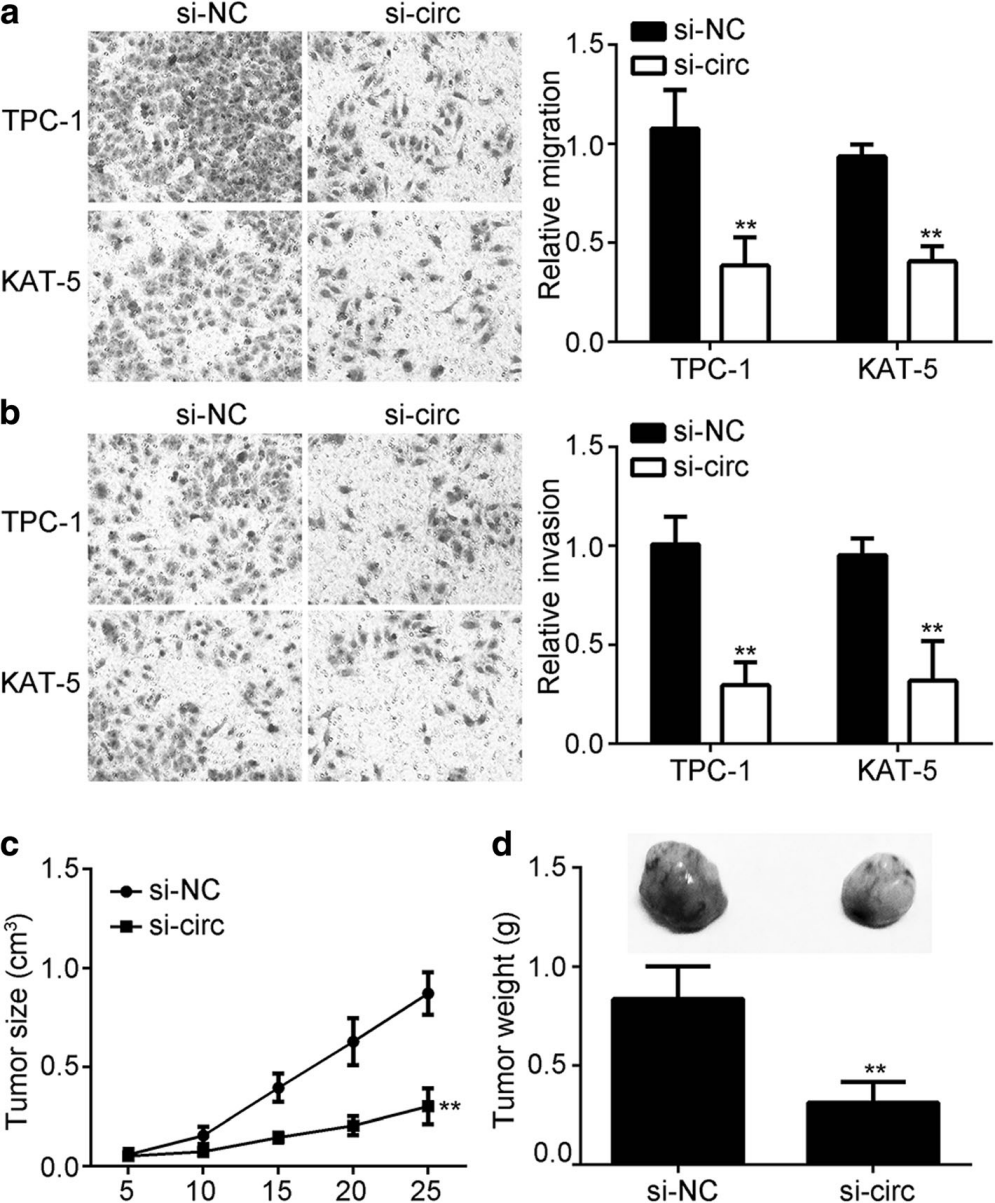
CircRNA_102171 knockdown suppresses migration and invasion, and reduces tumor growth in vivo. **a**, **b** Transwell assay showed that circRNA_102171 knockdown led to reduced cell number of migration and invasion. **c** CircRNA_102171 knockdown led to decreased tumor volume. **d** CircRNA_102171 knockdown reduced tumor weight. Si-NC: siRNA negative control. si-circ: siRNA targeting circRNA_102171. **p < 0.01

Metastasis is a major risk factor of PTC recurrence. Thus, the effect of circRNA_102171 on metastasis was assessed using Transwell assay. Silencing of circRNA_102171 significantly reduced the cell numbers of migration and invasion (Fig. 3a and b). Furthermore, xenograft assay was used to test the effect of circRNA_102171 on PTC in vivo. As shown, circRNA_102171 knockdown significantly suppressed the tumor size at described time points (Fig. 3c). At the endpoint, tumor weights were measured. Results showed that circRNA_102171 silencing also markedly decreased the tumor weights (Fig. 3d).

### CircRNA_102171 interacts with CTNNBIP1

**Fig. 4:**
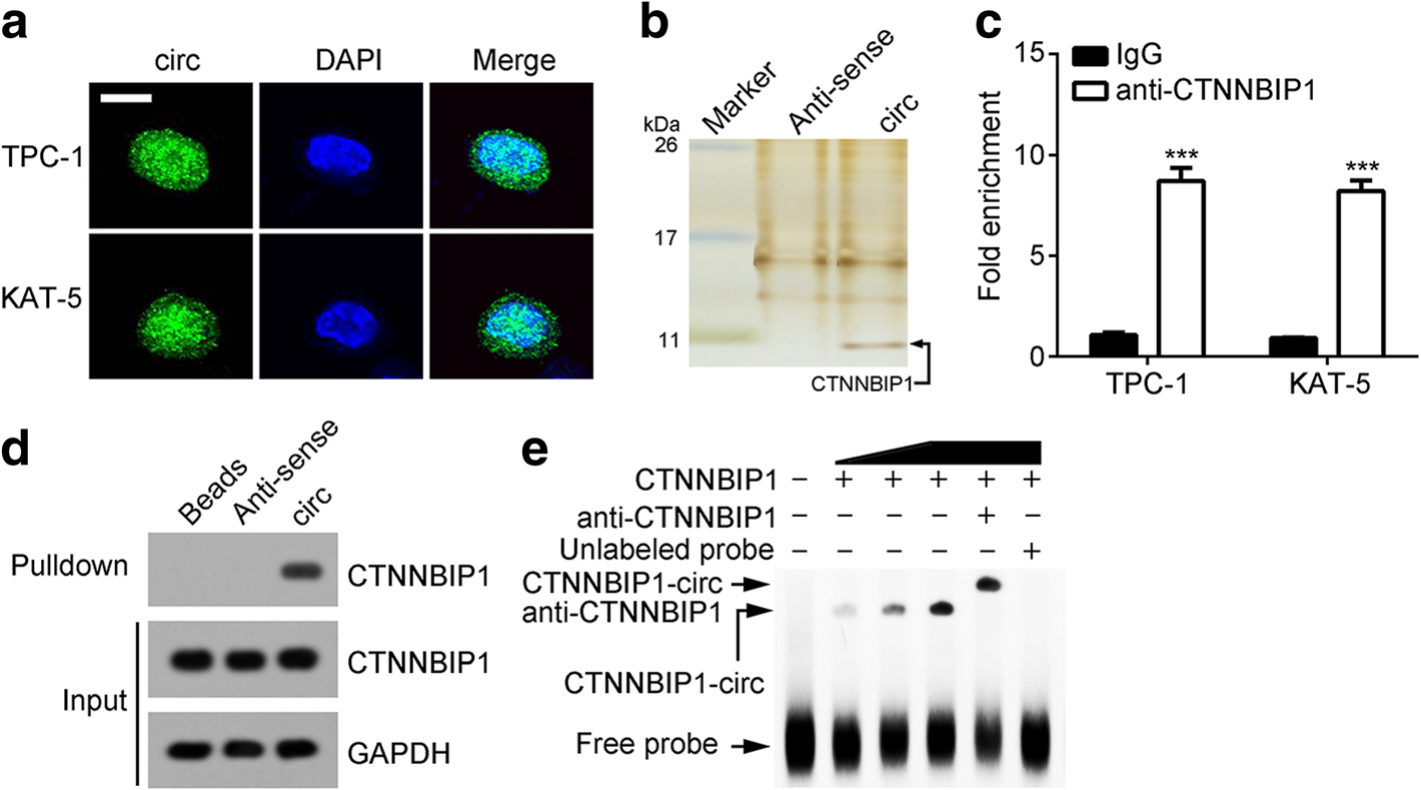
CircRNA_102171 interacts with CTNNBIP1. **a** RNA-FISH showed that circRNA_102171 was located in the nucleus of PTC cells. **b** CTNNBIP1 was a potential interactive protein of circRNA_102171 according to RNA-pulldown followed by silver staining and mass spectrum analysis. CircRNA_102171 and anti-sense were labeled with biotin. **c** RNA-IP showed that anti-CTNNBIP1 precipitated circRNA_102171 in cell lysates. **d** RNA-pull down showed that biotin-labeled circRNA_102171 precipitated CTNNBIP1 in cell lysates. **e** RNA-EMSA indicated that circRNA_102171 directly interacted with CTNNBIP1. ***p < 0.001

We then sought to investigate the mechanism of circRNA_102171. Firstly, we analyzed the localization of circRNA_102171 in PTC cells using RNA-FISH with biotin-labeled probes against circRNA_102171. As shown, circRNA_102171 was mainly located in the nucleus of TPC-1 and KAT-5 cells (Fig. 4a). We speculated that circRNA_102171 might interact with specific protein to exert function. Through RNA pulldown followed by silver staining and MS identification, we found that circRNA_102171 might interact with CTNNBIP1 (Fig. 4b). RNA IP assay using anti-CTNNBIP1 showed that CTNNBIP1 interacts with circRNA_102171 in TPC-1 and KAT-5 cells (Fig. 4c). RNA pulldown using biotin-labeled probes also indicated that circRNA_102171 precipitates CTNNBIP1 in TPC-1 cells (Fig. 4d). Moreover, RNA-EMSA assay illustrated that CTNNBIP1 interacts with circRNA_102171 directly (Fig. 4e). Above data together demonstrated that circRNA_102171 associates with CTNNBIP1 directly in PTC cells.

### CircRNA_102171 activates Wnt/β-catenin pathway through promoting β-catenin-TCF/LEF interaction

**Fig. 5:**
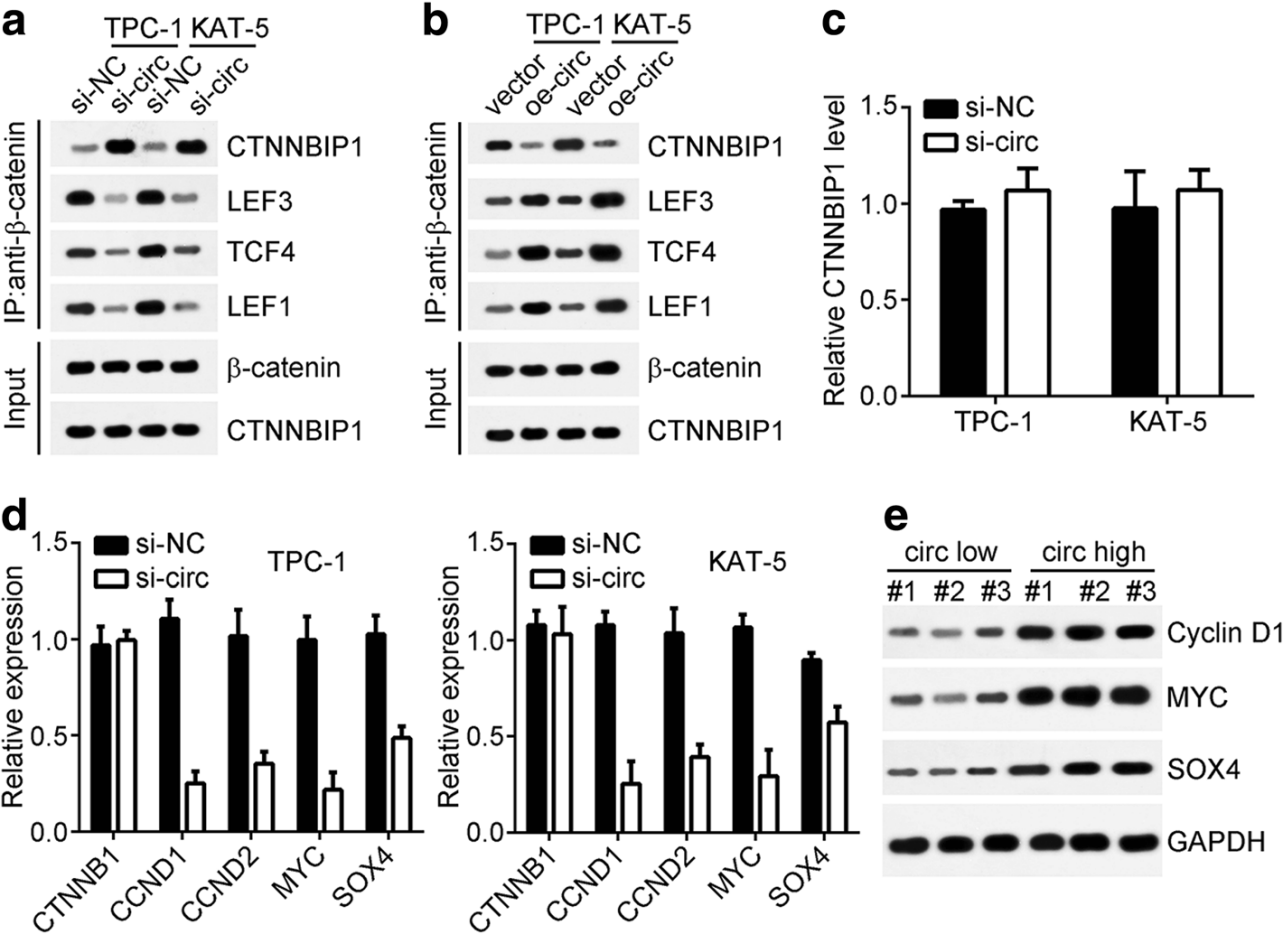
CircRNA_102171 activates Wnt/β-catenin pathway through promoting β-catenin-TCF/LEF interaction. **a** Western blot showed that circRNA_102171 knockdown inhibited β-catenin-TCF/LEF interaction while promoting CTNNBIP1/β-catenin interaction. **b** Western blot indicated that circRNA_102171 overexpression promotes β-catenin-TCF/LEF interaction while inhibiting CTNNBIP1/β-catenin interaction. **c** qRT-PCR showed that circRNA_102171 did not regulate CTNNBIP1 expression. **d** qRT-PCR showed that circRNA_102171 knockdown suppressed the expression of Wnt/β-catenin target genes (MYC/SOX4/CCND1/CCND2). **e** Western blot showed that higher expression of circRNA_102171 in PTC tissues was correlated with enhanced activation of Wnt/β-catenin pathway. Si-NC: siRNA negative control. si-circ: siRNA targeting circRNA_102171

CTNNBIP1 is β-catenin interacting protein and negatively regulates Wnt/β-catenin pathway through blocking the association of β-catenin with TCF/LEF [17]. Hence, we wondered whether circRNA_102171 regulates Wnt/β-catenin pathway through CTNNBIP1. Western blotting showed that knockdown of circRNA_102171 markedly attenuated the association of β-catenin with TCF/LEF while promoting the interaction between β-catenin and CTNNBIP1 (Fig. 5a). Moreover, circRNA_102171 overexpression achieved an opposite result (Fig. 5b). Notably, we found that circRNA_102171 could not affect the expression of CTNNBIP1 in PTC cells (Fig. 5c). Thus, circRNA_102171 regulates the interaction among CTNNBIP1, β-catenin and TCF/LEF in PTC cells. Then we analyzed the effect of circRNA_102171 on activation of Wnt/β-catenin. Through qRT-PCR, we found that circRNA_102171 knockdown significantly suppressed the expression of CCND1, CCND2, MYC and SOX4 (targets of Wnt/β-catenin) (Fig. 5d), indicating circRNA_102171 activates Wnt/β-catenin pathway in a CTNNBIP1-dependent manner. Moreover, we also observed that PTC samples with higher expression of circRNA_102171 showed enhanced activation of Wnt/β-catenin pathway (Fig. 5e).

### CTNNBIP1 suppresses PTC progression

**Fig. 6:**
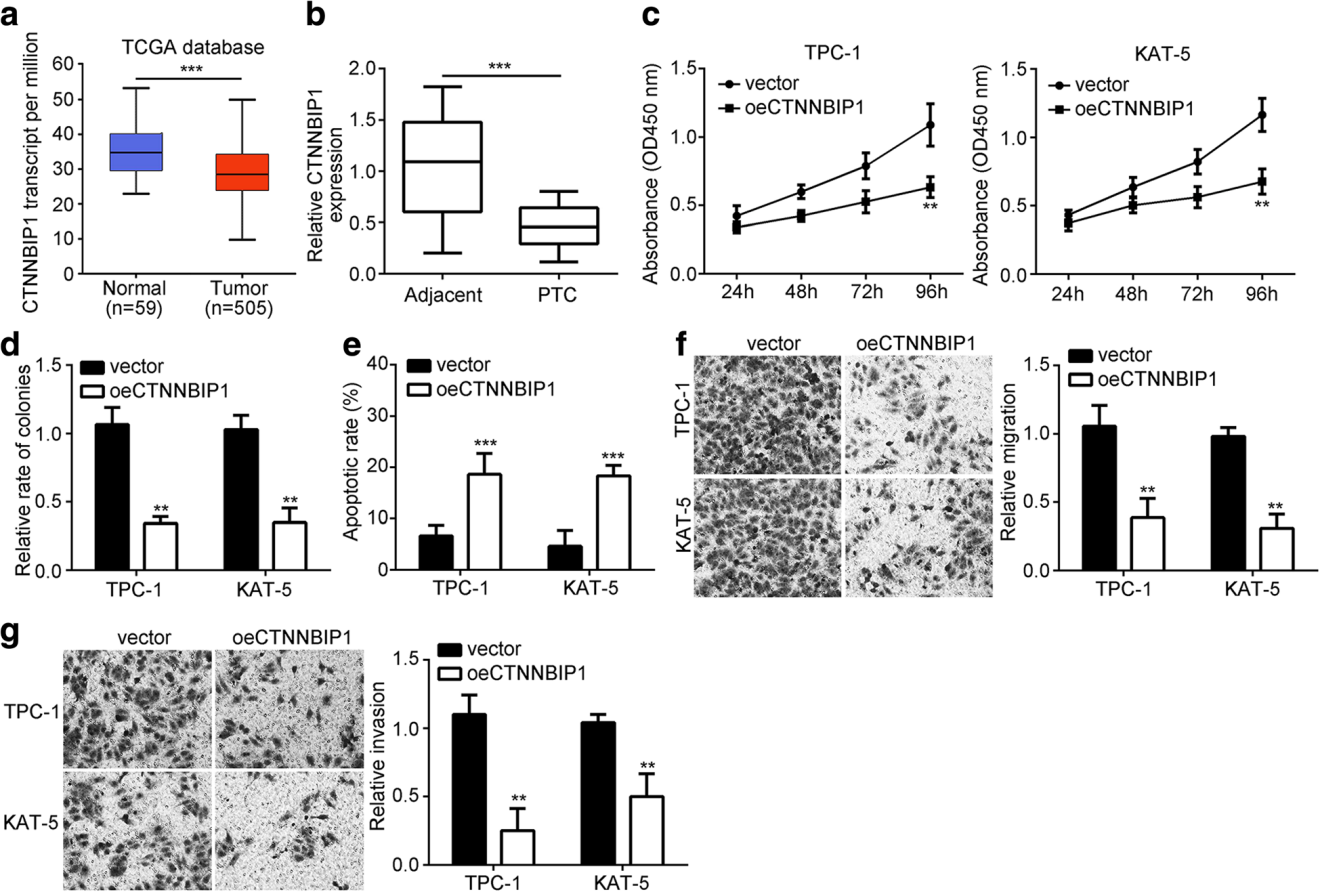
CTNNBIP1 suppresses PTC progression. **a** According to TCGA database, CTNNBIP1 level was downregulated in PTC tissues. **b** qRT-PCR analysis illustrated that CTNNBIP1 expression was decreased in PTC tissues. **c**, **d** CCK8 and colony formation assays indicated CTNNBIP1 overexpression suppressed PTC cell proliferation. **e** CTNNBIP1 overexpression promotes apoptosis. **f**, **g** Transwell assay showed that CTNNBIP1 overexpression inhibited migration and invasion. **p < 0.01 and ***p < 0.001

The role of CTNNBIP1 in PTC has not been revealed. And to better support that circRNA_102171 regulates PTC progression through CTNNBIP1, we investigated the effects of CTNNBIP1 in PTC. According to the TCGA database, we found that CTNNBIP1 expression was downregulated in PTC tissues (Fig. 6a). qRT-PCR analysis also validated it (Fig. 6b). Then we performed functional experiments. Through CCK8 and colony formation assays, we found that CTNNBIP1 overexpression significantly suppressed PTC cell proliferation (Fig. 6c and d). Yet, CTNNBIP1 ectopic expression promotes PTC cell apoptosis (Fig. 6e). Additionally, through Transwell assay, CTNNBIP1 overexpression significantly repressed the abilities of migration and invasion in PTC cells (Fig. 6f and g). These results demonstrated that CTNNBIP1 suppresses PTC progression.

## Discussion

As the most common cancer in endocrine system, PTC has developed into a challenge for public health. Sequencing studies have revealed many differentially expressed circRNAs in PTC tissues compared to paired normal tissues [18]. However, the roles of circRNAs in PTC remain largely unknown. Therefore, our study investigated the role of circRNA_102171. We demonstrated circRNA_102171 promotes PTC cell proliferation, migration and invasion while inhibiting apoptosis.

CircRNAs are often aberrantly expressed in cancer tissues and have been reported to regulate various human cancers, including breast cancer [12], bladder cancer [19], liver cancer [20], non-small cell lung cancer [8], oral squamous cell carcinoma [21] and cervical cancer [22]. For instance, Chen et al. reported that circ-ANAPC7 overexpression promotes acute myeloid leukemia development through targeting miR-181 family [23]. Yuan et al. showed that circ_0026344 upregulation predicts favorable prognosis and inhibits colorectal cancer development by targeting miR-21 and miR-31 [24]. Importantly, Wei et al. showed that circZFR promotes proliferation and metastasis of PTC cells through miR-1261/C8orf4 axis [13]. Wang et al. found that circ-ITCH inhibits PTC development by regulating miR-22-3p/CBL/β-catenin cascade [14]. Nevertheless, how circRNA_102171 functions in PTC remains unclear. Our study showed that circRNA_102171 level was elevated in PTC tissues. Through CCK8, colony formation and EdU incorporation assays, we demonstrated that circRNA_102171 promotes PTC cell proliferation, migration and invasion. Our study demonstrated that circRNA_102171 works as an oncogenic role in PTC, suggesting circRNA_102171 might be a potential therapeutic target.

Wnt/β-catenin signaling is a classical pathway involved in tumorigenesis. Aberrantly activation of Wnt/β-catenin pathway usually contributes to development of various cancers, such as gastric cancer [25], ovarian carcinoma [26], bladder cancer [27] and colorectal cancer [28]. Wnt/β-catenin is also reported to be important in PTC progression [14]. Wnt/β-catenin pathway is elaborately regulated by multiple factors in cancer. After association with TCF protein, β-catenin/TCF complex recognizes specific elements and initiates transcription [17]. Many molecular mechanisms are implicated in the regulatory process of Wnt/β-catenin pathway. For instance, APC degradation complex could promote the degradation of β-catenin, leading to inactivation of Wnt/β-catenin pathway [29]. lnc-β-Catm enhances the stability of β-catenin through promoting β-catenin methylation [30]. In our study, we reported a novel regulatory manner of Wnt/β-catenin activation. Through unbiased RNA-pulldown and MS identification, we screened that circRNA_102171 might interact with CTNNBIP1. According to RNA IP and pulldown assays, we demonstrated their interaction. Moreover, RNA-EMSA assay further demonstrated their direct interaction in PTC cells. CTNNBIP1 is β-catenin interacting protein. Evidences showed that CTNNBIP1 interacts with β-catenin to negatively regulate Wnt/β-catenin pathway by preventing formation of β-catenin/TCF complex [17]. The mechanism regulating the interaction between CTNNBIP1 and β-catenin remains vague. Our results showed that circRNA_102171 interacts with CTNNBIP1 and impaired the formation of CTNNBIP1/ β-catenin complex. Consequently, circRNA_102171 promotes the association of β-catenin with TCF proteins and activation of Wnt/β-catenin pathway.

Previous researches showed that CTNNBIP1 negatively regulates cancer progression [17, 31]. For example, CTNNBIP1 downregulation promotes progression of lung adenocarcinomas [32]. CTNNBIP1 upregulation inhibits glioma cell proliferation [33]. Whether CTNNBIP1 possess a similar role in PTC remains unknown. Through TCGA database and qRT-PCR analysis, we showed that CTNNBIP1 level was significantly downregulated in PTC tissues, suggesting a potential anti-tumor role. Furthermore, gain-of-function assays demonstrated that CTNNBIP1 overexpression suppressed the proliferation, migration and invasion of PTC cells. Our study reveals CTNNBIP1 is a tumor suppressor in PTC.

## Conclusion

In summary, our study confirmed that circRNA_102171 promotes the growth and invasion of PTC cells through activating Wnt/β-catenin pathway in a CTNNBIP1-dependent way. Therefore, our findings provide a new insight on the mechanism of PTC progression.

## Abbreviations

circRNA: Circular RNA
EMSA: Electrophoretic mobility shift assay
PTC: papillary thyroid cancer
RNA-FISH: RNA fluorescence in situ hybridization
